# Three-dimensional ultrasonographic features of diamniotic conjoined twins with body stalk anomaly

**DOI:** 10.1186/s12884-020-02920-0

**Published:** 2020-04-15

**Authors:** Guishuang Xiang, Yanting Wen, Li Zhang, Xiaoqian Tong, Lu Li

**Affiliations:** 1 Department of Ultrasound, The Fifth People’s Hospital of Chengdu, 33 Mashi Street, Chengdu, 611130 Sichuan Province China; 2 Department of Pathology, The Fifth People’s Hospital of Chengdu, Chengdu, 611130 China

**Keywords:** Conjoined twins, Monochorionic-diamniotic, Ultrasound, Yolk sac, Body stalk anomaly

## Abstract

**Background:**

Since conjoined twins were thought to be monoamniotic in the past, diamniotic conjoined twins would be improbable theoretically. Body stalk anomaly is a severe defect of the body wall, which is rare among twins. Body stalk anomaly in monochorionic diamniotic conjoined twins has never been reported prenatally so far as we know.

**Case presentation:**

Here we present an extremely rare case of concordant body stalk anomaly in monochorionic diamniotic conjoined twins. Ultrasonography at 9 + 5 weeks revealed one chorionic and two amniotic cavities, close apposition of abdomen, limited movement, and common umbilical vessels. Follow-up ultrasonography at 11 + 6 weeks and 13 + 2 weeks showed close apposition of the lower abdominal region with cystic structures and a small bowel-like mass between the two fetuses. Three-dimensional ultrasonography assisted in observing the entire appearance of both twins in earlier first trimester, including amnioticity, conjoined region and umbilical vessels. We attribute this diamniotic conjoined twin in our case to the fusion theory. A single yolk sac was observed, challenging the idea that yolk sac number predicts amnionicity. Identification of single yolk sac and its allantois may form a common body stalk during this fusion, leading to concordant body stalk anomaly in monochorionic diamniotic twins.

**Conclusions:**

Our case may provide important insights into both ultrasonographic features and embryogenesis of this extremely rare anomaly.

## Background

Monochorionic diamniotic (MD) conjoined twins is rare, and only 9 previous cases were reported [1–9]. This anomaly manifests on ultrasonography as one chorionic and two amniotic cavities, union of peritoneal cavities through an abdominal wall defect, conjoined intestine, and anorectal malformation. In the literature, fusion and fission models have been proposed to explain the embryogenesis of MD conjoined twins. The ultrasonographic features in our case are more consistent with the fusion hypothesis, which stipulates that the infraumbilical abdominal wall forms in the area of allantois and the caudal part of yolk sac. Fusion in this area disturbs the development of the infraumbilical abdominal wall and induces conjoining of adjacent intestines, leading to MD conjoined twins. Body stalk anomaly (BSA) is a severe defect of the body wall, which occurs in approximately 1 per 7500 pregnancies [10] in the first trimester. BSA is rare among twins, in which case it can be discordant or concordant. Here we describe a concordant BSA in MD conjoined twins treated at our hospital. We compare our case with similar ones in the literature to establish characteristic ultrasonography features that may facilitate early prenatal diagnosis, and we attempt to gain insights into the embryogenesis of these anomalies.

## Case presentation

**Fig. 1:**
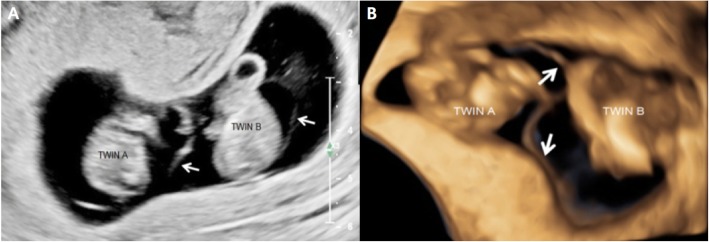
Diamniotic membrane (arrows) between the twins at 9 + 5 weeks in two-dimensional (**a**) and three-dimensional (**b**) ultrasonography, respectively

**Fig. 2:**
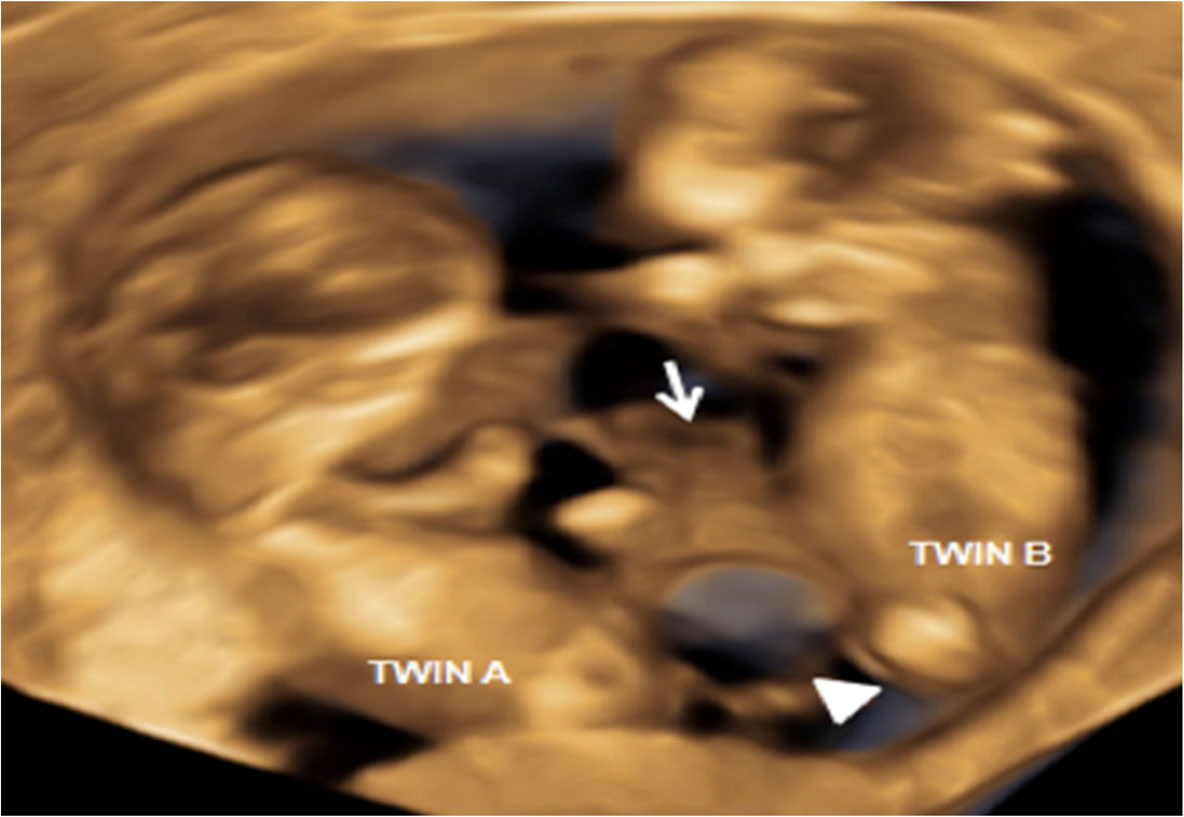
Cystic structures (arrow head) and bowel-like mass (arrow) between the two fetuses at 11 + 6 weeks in three-dimensional ultrasonography

A 27-year-old woman (gravida 1, para 0) who had become pregnant naturally was referred to our department at 9 + 5 weeks of gestation. She had no significant history of health issues and no history of multiple gestations. Two-dimensional (2D) and three-dimensional (3D) ultrasonography at 9 + 5 weeks revealed one gestational sac and an apparently dividing amniotic membrane surrounding each twin (Fig. 1a, b), which led to diagnosis of MD conjoined twin pregnancy. Only one yolk sac was observed (Fig. 1a). It was difficult to determine whether the abdominal structures were conjoined because of the twins’ relatively fixed position and limited movement. 2D/3D Ultrasonography at 11 + 6 weeks showed close apposition of the lower abdominal region with cystic structures and a small bowel-like mass between the two fetuses (Fig. 2), and limited fetal movements for both twins. Doppler ultrasound showed no free-floating umbilical cords for either twin, but several umbilical vessels coiled around the cystic structures and inserted into the placenta along the dividing membrane. Follow-up ultrasonography at 13 + 2 weeks of gestation revealed the twins were in the same relative position from all angles and moved together, the cystic structures and bowel-like mass seemed to lie in the exocoelom between the two amniotic cavities, and hydrodermia in twin B. The bladder configuration of either fetus could not be observed. In the end, MD conjoined twins with BSA was diagnosed. The parents requested induced abortion at 13 + 4 weeks of gestation and consent to pathological analysis.

**Fig. 3:**
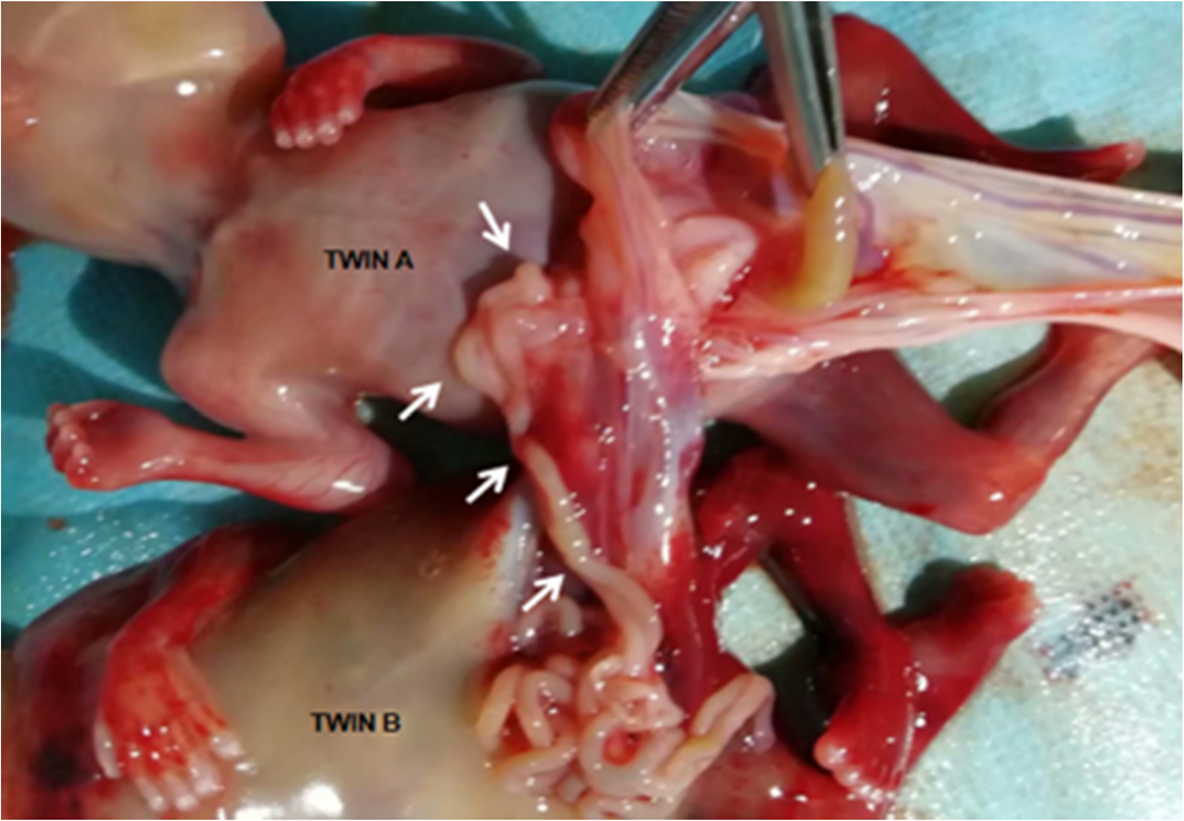
Post-abortion examination showed the intestinal tract of twin A was fused to that of twin B (arrows)

Post-abortion examination showed a single placenta with dividing amniotic membranes attached to the center of the placenta. Four umbilical vessels traveled along the dividing amniotic membrane and inserted together into the placenta. Both twins had gastroschisis with conjoined intestine and a fused skin bridge in lower part of abdomen. Twin B was found to have hydrodermia, strephenopodia and a small intestine exstrophy fused with the small intestine of twin A (Fig. 3). Twin A was found to have bowel involving the small intestine and colon herniated into exocoelom. An urachal remnant was also found outside the lower part of the abdomen. This postabortion analysis confirmed the diagnosis of MD conjoined twins with BSA.

## Discussion and conclusions

In the literature, MD conjoined twins are characterized by one chorionic and two amniotic cavities, the union of peritoneal cavities through an abdominal wall defect, conjoined intestine, and anorectal malformation [1–9]. 5 out of 9 cases were described as shared or bifurcated umbilical cord, 3 out of 9 cases were separated, and 1 out of 9 cases was not mentioned. Furthermore, 5 out of 9 previous cases were described as a single yolk sac, and the rest were not mentioned (Table 1). This case was diagnosed as MD conjoined twins because of the conjoined intestine and fused skin bridge in lower part of abdomen.

**Table 1:** Clinical characteristics of diamniotic conjoined twins

Study	Yolk sac no.	Twin characteristics	Umbilical cord	Gestational age, wk
Kapur R P 1994 [1]	single	Conjoined bowels in communicating omphalocele sac, shared persistent cloaca	Two separate umbilical cords, each containing 2 arteries and 1 vein	16
Costa S L 2006 [6]	NR	Conjoined bowels in communicating omphalocele sac, a shared bladder, anal atresia	NR	NR
Karnak I 2008 [4]	NR	Conjoined bowels in communicating omphalocele sac, cloacal anomaly	Two separate umbilical cords, each containing 3 vessels	NR
Tihtonen K 2009 [5]	NR	Conjoined bowels in communicating omphalocele sac	Shared umbilical cord with 4 arteries and 2 veins	18
Destephano C C 2010 [7]	single	Joined in abdominal region only (omphalopagus)	A bifurcated umbilical cord	11
Weston P J 2010	single	Conjoined bowels in communicating omphalocele sac, anal atresia, hypoplasia	Shared umbilical cord with 2 separate sets of blood vessels	After birth
Wielgos M 2014 [2]	NR	Conjoined bowels in communicating omphalocele sac	Two separate umbilical cords	27
Maruyama H 2015 [8]	single	Conjoined bowels, anal atresia	Shared short umbilical cord	12
Nupur Shah 2019 [9]	single	Joined at the periumbilical region	A bifurcated umbilical cord	8

*NR* Not reported

BSA is a severe abdominal wall defect and is associated with abnormal embryonic folding in the 5th week of gestation [11]. It seems to be more common in twin pregnancies. Previous reports showed a large abdominal wall defect and herniation of the liver, bowels, or heart into the exocoelom [12–14]. However, none of the cases showed conjoined intestine or fused skin bridge, in contrast to the present case. Gastroschisis and intestine partly herniated into exocoelom in our case are consistent with characteristics of BSA.

3D ultrasonography assisted in revealing the entire appearance of both twins, including amnioticity, conjoined region and the location of umbilical vessels in the first trimester. However, this may not always prove feasible early in the first trimester, which may show limited fetal movement and unclear position of the herniated abdominal mass, as in our case. The diagnosis of BAS in MD conjoined twins was not clear until we found the bowel-like mass lying in the exocoelom at 13 + 2 weeks.

**Fig. 4:**
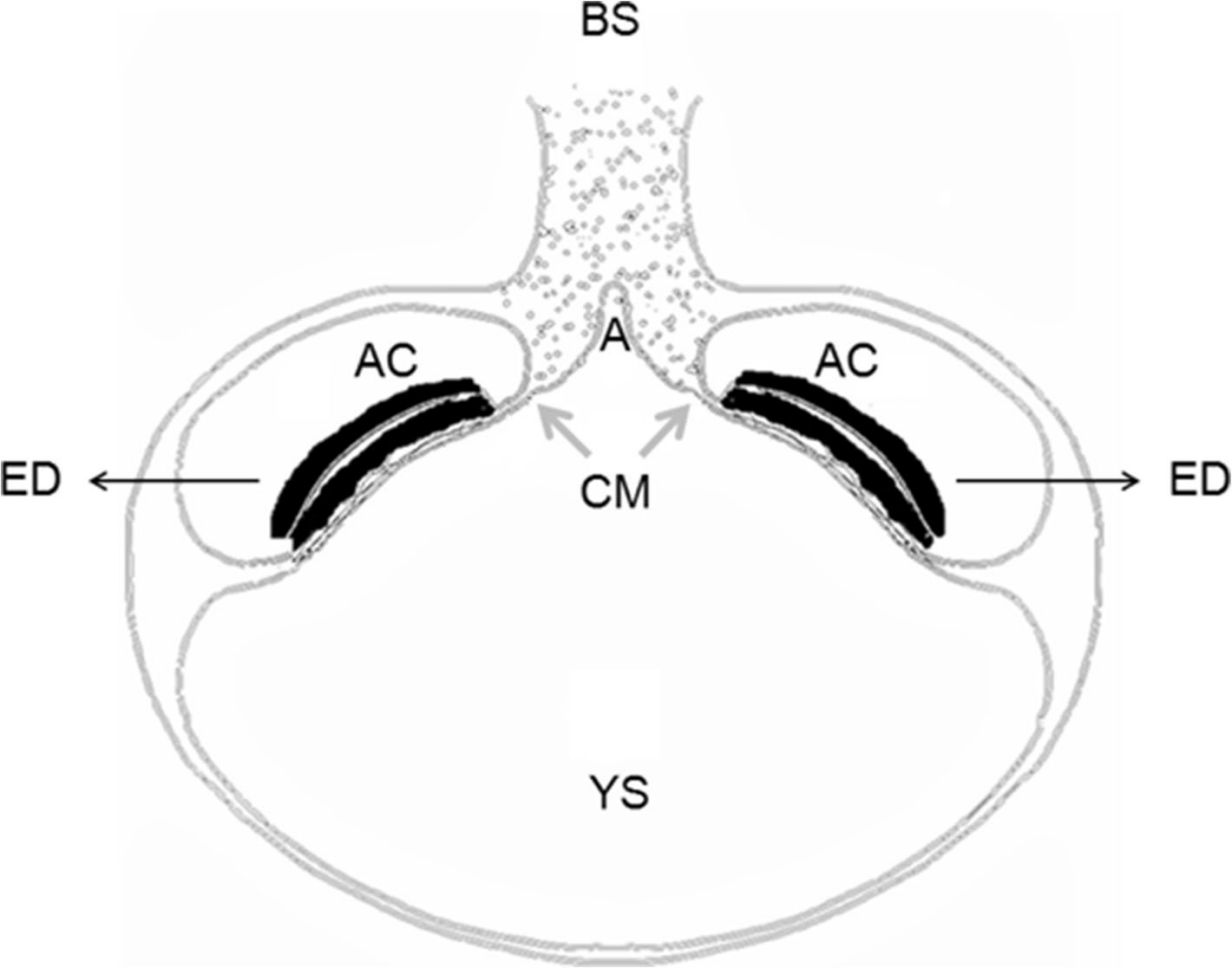
Drawing of the embryogenetic model to explain our case. The common body stalk and single allantois contrast with the separated body stalk and two allantoises of other embryogenetic models. A, allantois; AC, amniotic cavity; BS, body stalk; CM, cloacal membrane; ED, embryonic disk; YS, yolk sac

Fission and fusion models have been proposed to explain the embryogenesis of MD conjoined twins. The fission hypothesis [1] speculates that there is only one amniotic cavity with two embryos conjoined at specific sites. During embryo folding, a crease develops between the two embryos, and the amniotic cavity is divided into two. This hypothesis cannot explain gastroschisis and urachal remnant at the lower part of the abdomen visible by ultrasonography. The fusion hypothesis [15] stipulates that the infraumbilical abdominal wall forms in the area of allantois and the caudal part of yolk sac. Fusion in this area disturbs the development of the infraumbilical abdominal wall and induces conjoining of adjacent intestines, leading to MD conjoined twins. Our case had a single yolk sac, which may provide insights into how those anomalies developed. In the literature, yolk sac number can predict amnionicity [16], however, a single yolk sac in MD conjoined twins may be associated with a higher risk of congenital defects [17]. Shen et al. [18] speculate that yolk sac formation occurs before the differentiation of amnion and after that of chorion. They suggested that the splitting occurs after chorion and before amnion differentiation. As a consequence, only one yolk sac develops, with one chorionic and two amniotic cavities. We speculate that the single yolk sac in our case explains the single allantois, since the allantois is a small diverticulum of the caudal part of the yolk sac. We hypothesize that the single yolk sac and its allantois induce this fusion [15] and form common body stalk (Fig. 4). This may indicate common umbilical vessels and herniated intestines in the exocoelom, suggesting a BSA in MD conjoined twins.

Considering the uniformly fatal nature of MD conjoined twins with BSA, early prenatal diagnosis is critical for averting complications during pregnancy. Additional work is needed to explore the mechanisms behind this anomaly and to understand its epidemiology and risk factors.

## Data Availability

Not applicable.

## Abbreviations

2D: Two-dimensional
3D: Three-dimensional
MD: Monochorionic diamniotic
BSA: body stalk anomaly
